# IMPULSE OSCILLOMETRY AND SPIROMETRY IN SCHOOLERS SUBMITTED TO THE SIX-MINUTE WALK TEST

**DOI:** 10.1590/1984-0462/;2018;36;4;00007

**Published:** 2018

**Authors:** Maíra Seabra de Assumpção, José Dirceu Ribeiro, Renata Maba Gonçalves Wamosy, Paloma Lopes Francisco Parazzi, Camila Isabel Santos Schivinski

**Affiliations:** a Universidade Estadual de Campinas, Campinas, SP, Brasil.; b Universidade do Estado de Santa Catarina, Florianópolis, SC, Brasil.

**Keywords:** Child, Oscillometry, Respiratory function tests, Effort test, Criança, Oscilometria, Teste de função respiratória, Teste de esforço

## Abstract

**Objective:**

To verify repercussions of submaximal exercise testing on respiratory mechanics and pulmonary function in schoolchildren.

**Methods:**

Cross-sectional study, with children aged 7 to 14 years, who had their respiratory mechanics assessed by impulse oscillometry (IOS), and pulmonary function by spirometry. They performed the six-minute walk test (6MWT), as per the standards by the American Thoracic Society. The 6MWT was performed twice with a 30-minute interval. IOS and spirometry were performed before the first 6MWT (Pre-6MWT) and immediately after the first (Post-6MWT_1_) and second walking tests (Post-6MWT_2_). The results in these three phases were compared by analysis of variance for repeated measures (post-hoc Bonferroni test) or by the Friedman’s test, with p≤0.05 considered significant.

**Results:**

Twenty-one subjects participated in the study: 53% were males and mean age was 10.9±2.3 years. There were differences between total resistance (R5) and central airway resistance (R20) at the three phases of assessment (p=0.025 and p=0.041, respectively). Post-hoc analysis indicated increase in R5 when Pre-6MWT and Post-6MWT_1_ were compared (R5=0.540±0.100 *versus* 0.590±0.150 kPa/L/s, p=0.013; and R20=0.440±0.800 *versus* 0.470±0.100 kPa/L/s, p=0.038). Forced expiratory flow 25-75% (FEF_25-75%_) changed over time (p=0.003).

**Conclusions:**

Repercussions were: increase in central and total airway resistance and reduction of FEF_25-75%_ after 6MWT in schoolchildren, suggesting that greater attention should be given to submaximal tests in children with predisposition to airways alterations.

## RESUMO

**Objetivo:**

Verificar as repercussões do teste de esforço submáximo na mecânica respiratória e na função pulmonar de escolares.

**Métodos:**

Estudo transversal com escolares de 7 a 14 anos submetidos à avaliação da mecânica respiratória por sistema de oscilometria de impulso (IOS) e da função pulmonar pela espirometria. Realizou-se também o teste de caminhada de seis minutos (TC6), todos segundo os padrões da Sociedade Torácica Americana. O TC6 foi executado duas vezes com intervalo de 30 minutos entre cada teste. O IOS e a espirometria foram feitos antes do primeiro TC6 (pré-TC6) e repetidos imediatamente após o primeiro TC6 (pós-TC6_1_) e após o segundo TC6 (pós-TC6_2_). A comparação dos resultados nos três tempos do estudo se deu por análise de variância para medidas repetidas (teste *post-hoc* de Bonferroni) ou teste de Friedman, sendo significante p≤0,05.

**Resultados:**

Participaram 21 sujeitos; 53% masculinos e idade média de 10,9±2,3 anos. Encontraram-se diferenças entre resistência total (R5) e resistência central das vias aéreas (R20) nos 3 tempos do estudo (p=0,025 e p=0,041, respectivamente). A análise *post-hoc* indicou aumento de resistência R5 entre pré-TC6 e pós-TC6_1_ (R5=0,540±0,100 *versus* 0,590±0,150 kPa/L/s, p=0,013; e R20=0,440±0,800 *versus* 0,470±0,100 kPa/L/s, p=0,038). A única variável espirométrica com alteração no decorrer do tempo foi o fluxo expiratório forçado 25-75% (FEF_25-75%_) (p=0,003).

**Conclusões:**

As repercussões encontradas foram: aumento da resistência total e da resistência central das vias aéreas e redução do FEF_25-75%_ após o TC6 em escolares, sugerindo a necessidade de mais atenção na realização de testes submáximos em crianças com alguma predisposição a alterações das vias aéreas.

## INTRODUCTION

The six-minute walk test (6MWT) is among the main functional capacity assessment tests used in pediatrics that are considered low-cost, easy to use, reliable and reproducible.^1^ It is a submaximal test capable of reflecting the limitations of individuals with chronic respiratory diseases^2^ ^,^ ^3^ which is commonly used to evaluate children^1^ ^,^ ^4^ with the purpose of establishing reference values/equations^5^ ^,^ ^6^ and investigating respiratory, neuromuscular and skeletal diseases.^7^ Well established in the literature, it follows guidelines and recommendations by the American Thoracic Society (ATS) and the European Respiratory Society (ERS).^8^ ^,^ ^9^

Although this is a physical exercise testing and the literature reports possible changes in airways’ caliber as an important determinant of airflow and respiratory efforts during physical activities,^10^ ^,^ ^11^ little is known about the dynamic relationship between airway resistance and its execution. According to the ATS,^12^ exercise-induced bronchospasm is the acute airway narrowing in response to exercise that happens even in patients without asthma diagnosis. Research in this field has focused on the relationship between bronchial narrowing, pulmonary function and cardiorespiratory and physiological parameters in individuals with asthma.^10^ ^,^ ^11^

In asthma and other respiratory diseases, spirometry has been routinely indicated for monitoring and intervention control. It is an exam conducted within well-defined guidelines and considered the gold standard to identify airway obstruction and restriction in pulmonary diseases.^13^ Due to its difficult execution in the pediatric age group, impulse oscillometry system (IOS) is an important tool to analyze pulmonary mechanics parameters, as it is less complex to execute^14^ and complements spirometric results.^15^ IOS evaluates impedance (Z), resistance (R) and reactance (X5) parameters in multiple frequencies, from 5 to 35 Hertz, based on breaths at tidal volume. Described as a practical exam for being fast-paces and not requiring expiratory effort by individuals, it allows the identification of obstructive changes in the respiratory system.^16^ ^,^ ^17^

The literature has reports on investigation of relationships between respiratory diseases, such as asthma and cystic fibrosis, and oscillometric/spirometric parameters; however, the evaluation along with functional tests has not been done yet. Individuals with respiratory diseases are routinely assessed for their functional capacity; nevertheless, possible changes following exercise testings are not monitored. Both aspects of evaluation - functional capacity, by exercise testings such as the 6MWT, and respiratory integrity, by IOS and spirometry - are part of the handling of chronic diseases. Knowing some of the relationships between them in healthy children favors the understanding of normal cardiorespiratory mechanisms and responses, making comparisons and the control of sick children more feasible. In this line, evaluating the repercussion of a submaximal exercise test like the 6MWT in the airways of healthy children and adolescents seems relevant.

The 6MWT has been indicated for the adult and pediatric populations to evaluate functional capacity, in epidemiological research proposals, monitoring of interventions effectiveness, and especially as a parameter for response to pulmonary rehabilitation programs and follow-up of physiotherapeutic protocols.^18^ ^,^ ^19^ Its repercussion in parameters of non-invasive, easy-to-perform, safe and validated methods of evaluation for the pediatric population, such as IOS and spirometry, has been poorly investigated yet. Thus, to date, there are no studies evaluating the repercussion of a submaximal exertion test on IOS and spirometry variables in children and adolescents. That being said, the purpose of this study was to verify the behavior of respiratory mechanics and pulmonary function in schoolchildren submitted to 6MWT.

## METHOD

This is a cross-sectional study with schoolchildren and approved by the Research Ethics Committee of *Universidade do Estado de Santa Catarina* (UDESC), under protocol 99/2011, conducted in Florianópolis (Santa Catarina, Brazil). Data was collected in public and private institutions of primary and secondary education in the Greater Florianópolis region from October 2012 to December 2014. Children’s caregivers/guardians were informed about the study and, upon agreeing to participate, they signed the informed consente form and fulfilled the ISAAC questionnaire’s (International Study of Asthma and Allergies in Childhood) modules on asthma and rhinitis, in the version validated for the Brazilian Portuguese language.^20^ ^,^ ^21^

The sample was assembled by selection of participants in the study to determine reference equations for IOS among healthy Brazilian children and adolescents.^22^ This study included schoolchildren of both sexes, aged between 6 and 14 years, no history of prematurity or passive exposure to smoking, no respiratory infection up to two weeks prior to evaluations and no respiratory impairment such as asthma and allergic rhinitis, according to the cut-off scoring proposed by ISAAC:

- asthma module: ≥5 points for children aged 6 to 9 years; ≥6 points for children aged 10 to 14 years)^20^ ^,^ ^23^;
- Allergic rhinitis module: ≥4 points for children aged 6 to 9 years; ≥3 points for children aged 10 to 14 years).^20^ ^,^ ^21^

Children who failed to perform all stages of the assessment properly were excluded.

Weight was measured on a digital glass scale Ultra Slim W903 Wiso^®^ (Santa Catarina, Brazil), and height by a portable stadiometer Sanny^®^ (São Paulo, Brazil). Body mass index (BMI) was defined according to the percentile curves proposed by the World Health Organization (WHO) (http://www.telessaudebrasil.org.br/apps/calculadoras/page=7).^24^

All participants underwent forced expiratory maneuvers of spirometry by the Jaeger^TM^ MasterScreen^TM^ IOS (Erich Jaeger, Germany) (spirometry module), as recommended by both ATS and ERS.^13^ In order to avoid excessive maneuvers, which could interfere with results interpretation, each participant made three or four forced expiratory maneuvers, respecting the criteria of three acceptable maneuvers and two reproducible maneuvers.

Respiratory mechanics was analyzed in the IOS Jaeger^TM^ MasterScreen^TM^ system (Erich Jaeger, Germany) (impulse oscillometry module) coupled to the same equipment, as per ATS/ERS standards.^25^ ^,^ ^26^ Children were instructed to spontaneously breathe at tidal volume, with the mouth engaged in a mouthpiece. Recording time for data acquisition was set between 20 and 30 seconds, with three measurements each. The best measure of these three was considered, complying with the criteria of maneuverability and 10% minimum difference between their parameters. The following parameters were taken into account: total (R5) and central (R20) respiratory resistance; respiratory reactance (X5), respiratory impedance (Z5), resonance frequency (Fres), and reactance area (AX).

Functional capacity was assessed with the 6MWT, conducted according to recommendations of ATS^8^ in a 30-meter flat covered corridor. Polar Fs2c BLK^®^ digital timer (Kempele, Finland) was used, as well as Nonin Onyx 9500^®^ digital oximeter (Minnesota, USA) to measure heart rate (fc) and peripheral oxygen saturation (SpO_2_), and Prestige Medical^®^ sphygmomanometer (California, United States) to measure blood pressure (BP). Dyspnea sensation was measured by the modified Borg scale. Values proposed for the distance covered were based in the equation of Priesnitz et al.^18^ for Brazilian children.

**Figure 1:**
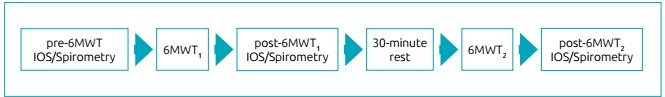
Flowchart of the sequence of procedures taken to evaluate repercussions of the six-minute walk test on respiratory mechanics and lung function in healthy schoolchildren.

IOS parameters and spirometry were analyzed in resting conditions, that is, before the first 6MWT (pre-6MWT), immediately after the first 6MWT (post-6MWT_1_) and after the second 6MWT (post-6MWT2), as shown in Figure 1. In addition to hygiene for current research, the final sample was required to hold children and adolescents who had not exceeded the total number of 12 forced expiratory maneuvers in spirometry considering the three stages of evaluation (pre-6MWT, post-6MWT_1_ and post-6MWT_2_).

6MWT: six-minute walk test; IOS: impulse oscillometry system; pre-6MWT: before six-minute walk test; post-6MWT1: after first six-minute walk test; post-6MWT2: after second six-minute walk test; 6MWT1: first six-minute walk test; 6MWT2: second six-minute walk test;

To calculate sample size, we considered a pilot study involving healthy children aged 7 to 14 years. Due to its representativeness in total mechanical load of the respiratory system, the variable selected to check for changes in a more specific way was respiratory impedance. Participants had mean pre-6MWT_1_ of 0.5 kPa/L/h and standard deviation of 0.11 kPa/L/s and post-6MWT_2_ of 0.6 kPa/L/s, with standard deviation of 0.15 kPa/L/s, with difference detected of 0.1 kPa/L/s. Significance level was set at 5%, with test power of 80%. With these estimates, the adequate sample should allocate 19 individuals.

Data were nalyzed in the Statistical Package for the Social Sciences^®^ (SPSS) software 20.0 (IBM, New York, USA). Initially, we verified data distribution by means of the Shapiro Wilk test and then applied the ANOVA (post-hoc Bonferroni) test for repeated measures or the Friedman’s test to compare results in the three phases of assessment. Significance was set at 5% (p≤0.05).

## RESULTS

Of total 864 children and adolescents evaluated in the study by Assumpção et al.^22^, in which 123 individuals participated, 21 schoolchildren (10 boys) were able to perform the spirometric maneuvers according to preestablished inclusion criteria and constituted the final sample.

The students’ age ranged from 7 to 14 years, with mean 10.9 years. There were no participant losses during tests (intercurrences and/or withdrawals), and all students met the criteria determined for the present study.

Descriptive data regarding weight, height, BMI and distance traveled in the 6MWT are shown in Table 1. Complying with WHO classification, the sample consisted of 21 children, 14 being classified as eutrophic (adequate weight) and seven as non-eutrophic (four overweight and three obese). There were no differences between baseline cardiorespiratory parameters of spirometric variables and oscillometric parameters in the study.

**Table 1:** Mean and standard deviation of anthropometric variables.

Anthropometric variables	Mean±standard deviation	Median (min-max)
Weight (kg)	41.1±10.8	42.9 (23.9-59.0)
Stature (cm)	147.9±12.3	147.2 (126.8-167.0)
BMI (kg/m^2^)	18.4±2.8	18.5 (13.2-25.3)

Cm: centimeters; kg: kilogram; BMI: body mass index; kg/m^2^: kilogram per square meter.

Only nine children performed better than expected when considering reference values for Brazilian children. The description of cardiorespiratory parameters for the first and the second 6MWT is found in Table 2.

**Table 2:** Description of cardiopulmonary parameters of first and second six-minute walk tests.

Parameters assessed	6MWT_1_		6MWT _2_	
	Basal	Final	Basal	Final
BP (mmHg)	99/59	106/64	101/61	107/63
HR (bpm)	81.1±13.5 81 (55-105)	128.5±22.7 131 (97-166)	84.4±14.1 86 (62-108)	125.3±22.5 126 (81-162)
SpO_2_ (%)	98.8±0.3 99 (98-99)	98.6±0.7 99 (96-99)	98.8±0.5 99 (97-99)	98.6±0.6 99 (97-99)
RR (bpm)	19.3±2.8 20 (15-24)	25.5±4.4 24 (19-36)	18.7±2.8 20 (14-24)	26.4±4.6 24 (18-36)
Borg	0 0	0.4±0.7 0 (0-3)	0 0	0.4±0,9 0 (0-4)
DT (m)	583.3±100.5 600.4 (545.2-659.4)		605.1±92.6 594.7 (439.6-779.6)	
Speed (m/s)	1.6±0.2 1.6 (0.9-2.0)		1.6±0.2 1.6 (1.2-2.1)	

6MWT: six-minute walk test; 6MWT_1_: first six-minute walk test; 6MWT_2_: second six-minute walk test; BP (mmHg): blood pressure, in millimeters of mercury; HR (bpm): heart rate, in beats per minute; SpO_2_(%): peripheral oxygen saturation, in percentage; RR (bpm): respiratory rate, in breaths per minute; DT (m) distance traveled, in meters; Speed (m/s): speed, in meters per second. Values expressed as mean and standard deviation; minimum and maximum.

After the two tests, a difference between parameters of total airway resistance (R5) and central airway resistance (R20) (p=0.041 and p=0.025, respectively) was observed (Table 3). Significant increase in R5 was observed after post-hoc analysis (R5: 0.540±0.110 *versus* 0.590±0.150 kPa/L/s, p=0.013), as well as R20 (0.440±0.800 *versus* 0.470±0.100 kPa/L/s, p=0.038). The increase in R20 was also significant when comparing pre-6MWT and post-6MWT_2_ (0.440±0.800 *versus* 0.470±0.110 kPa/L/s, p=0.034).

**Table 3:** Analysis of oscillometric parameters Pre-6MWT, Post-6MWT_1_ and Post-6MWT_2_.

Oscillometric parameters	Pre-6MWT Mean±SD Median (min-max)	Post-6MWT_1_ Mean±SD Median (min-max)	Post-6MWT_2_ Mean±SD Median (min-max)	p-value
R5 (Kpa/L/s)	0.54±0.11^I^ 0.58 (0.35-0.88)	0.59±0.15^I^ 0.62 (0.03-1.24)	0.57±0.15 0.59 (0.31-1.15)	0.025*
R20 (Kpa/L/s)	0.44±0.08^I.II^ 0.45 (0.30-0.65)	0.47±0.10^I^ 0.48 (0.31-0.75)	0.47±0.11^II^ 0.48 (0.26-0.78)	0.041*
X5 (Kpa/L/s)	-0.14±0.05 -0.15 (-0.27-0.06)	-0.14±0.06 -0.43 (-0.31-0.05)	-0.14±0.05 -0.14 (-0.31-0.05)	0.830
Z5 (Kpa/L/s)	0.56±0.11 0.59 (0.35-0.93)	0.61±0.15 0.64 (0.34-1.29)	0.59±0.16 0.62 (0.31-1.19)	0.051
Fres (Hz)	16.39±4.44 16.87 (9.26-25.62)	17.10±4.87 17.66 (9.24-30.17)	16.89±5.36 17.18 (9.40-30.80)	0.854
Ax (Kpa/L/s)	0.80±0.40 0.77 (0.14-2.62)	0.89±0.58 0.87 (0.12-5.64)	0.88±0.66 0.78 (0.15-4.18)	0.810

6MWT: six-minute walk test; 6MWT_1_: first six-minute walk test; 6MWT_2_: second six-minute walk test; SD: standard deviation; min: minimum; max: maximum; * significance of the ANOVA test (post-hoc Bonferroni) for repeated measures (p≤0.05); ^I^significance between pre-6MWT and post-6MWT_1_; ^II^significance between pre-6MWT and post-6MWT_2_; R5: total resistance; R20: central resistance; X5: 5 Hz reactance; Z5: respiratory impedance at 5 Hz; Fres: resonant frequency; Hz: Hertz; AX: reactance area; Kpa/L/s: unit of measurement in Kilopascal per liter per second; Kpa/L: Kilopascal per liter.

The only spirometric variable that showed significant change after tests was forced expiratory flow between 25-75% (FEF_25-75%_) (p=0.003), identified in Friedman’s test. Multiple analysis was used to detail the timing of this change. This parameter was shown to decrease between pre and post-6MWT_1_ moments (85,900±19,900% *versus* 80,800±20,200%, p=0,010), as well as in pre and post-6MWT_2_. FEF_25-75%_ (Z score) also changed when compared to pre-6MWT and post-6MWT_2_ (p=0.001). In the present study, only four infants presented baseline FEF_25-75%_ values below 70% (Table 4).

**Table 4:** Analysis of spirometric parameters Pre-6MWT, Post-6MWT_1_ and Post-6MWT_2_.

Spirometric variables	Pre-6MWT Mean±SD Median (min-max)	Post-6MWT_1_ Mean±SD Median (min-max)	Post-6MWT_2_ Mean±SD Median (min-max)	p-value
FVC (L)	2.6±0.6 2.5 (1.7-4.0)	2.6±0.7 2.5 (1.6-4.1)	2.60±0.6 2.6 (1.8-4.0)	0.611
FVC %	97.6±6.6 97.5 (87.5-114.0)	97.4±7.4 98.2 (86.4-116.6)	96.6±8.7 95.7 (78.9-114.5)	0.551
FVC (Z score)	-0.1±0.6 -0.3 (-1.1-1.6)	-0.2±0.6 -0.3 (-1.2-1.8)	-0.2±0.7 -0.3 (-1.7-1.7)	0.764
FEV_1_ (L)	2.2±0.5 2.1 (1.5-3.5)	2.2±0.5 2.2 (1.5-3.3)	2.2±0.4 2.1 (1.5-3.0)	0.058
FEV_1_ %	90.4±5.4 89.6 (80.4-100.2)	88.8±5.7 87.8 (78.4-100.7)	87.4±6.1 86.8 (78.2-101.8)	0.053
FEV_1_ (Z score)	-0.3±0.5 -0.3 (-1.0-0.6)	-0.3±0.9 -0.6 (-1.2-3.0)	-0.5±0.6 -0.6 (-1.5-0.8)	0.200
PEF (L/s)	4.9±1.2 4.3 (2.9-7.5)	4.8±1.1 4.6 (2.6-7.4)	4.8±1.1 4.6 (2.4-7.5)	0.438
PEF %	85.3±11.9 81.7 (64.2-108.9)	82.6±10.8 81.6 (58.0-110.8)	83.6±11.2 82.1 (53.6-11.4)	0.105
PEF (Z score)	4.7±0.7 4.8 (3.4-6.3)	4.5±0.6 4.4 (3.4-6.3)	4.6±0.7 4.5 (3.0-6.4)	0.170
FEF_25-75%_ (L)	2.6±0.7 2.4 (1.3-4.7)	2.5±0.8 2.3 (1.2-4.3)	2.4±0.8 2.2 (1.4-4.8)	0.608
FEF_25-75%_ %	85.9±19.9^I.II^ 80.8 (55.5-136.7)	80.8±20.2^I^ 74.5 (52.0-125.2)	77.9±19.4^II^ 72.2 (44.0-120.3)	0.003*
FEF_25-75%_ (Z score)	-0.3±0.8^II^ -0.6 (-1.8-1.6)	-0.4±1.2 -0.9 (-2.2-3.3)	-0.6±1.2^II^ -1.1 (-2.9-2.2)	0.001*
FEV_1_/FVC	0.8±0.0 0.8 (0.7-0.9)	0.8±0.0 0.8 (0.7-0.9)	0.8±0.0 0.8 (0.7-0.9)	0.189
FEV_1_/FVC (Z score)	-0.2±0.9 -0.3 (-2.0-1.7)	-0.2±1.0 -0.1 (-2.3-2.3)	-0.4±0.8 -0.5 (-2.1-1.3)	0.079

6MWT: six-minute walk test; 6MWT_1_: first six-minute walk test; 6MWT_2_: second six-minute walk test; SD: standard deviation; min: minimum; max: maximum; *significance of Friedman’s test (p≤0.05); ^I^significance between pre-6MWT and post-6MWT_1_; ^II^significance between pre-6MWT and post-6MWT_2_; FVC (L): forced vital capacity in liters; FVC%: forced vital capacity in percentage; FEV_1_ (L): first-second forced expiratory volume in liters; FEV_1_%: first-second forced expiratory volume in percentage; PEF (L/s): forced peak expiratory flow in liters per second; PEF%: forced peak expiratory flow in percentage; FEF_25-75%_ (L): forced expiratory flow between 25-75% of FVC in liters; FEF_25-75%_: forced expiratory flow between 25-75% of FVC in percentage; FEV_1_/FVC ratio: Tiffeneau index.

## DISCUSSION

The analysis of respiratory mechanics behavior and lung function in schoolchildren undergoing submaximal exertion tests leads to the understanding of respiratory system changes. In particular, the present study demonstrated that the 6MWT changed the spirometric variable FEF_25-75%_. Considered an important measure of respiratory flows, it allows evaluation of airway permeability, since it represents the airflow speed exclusively from the bronchi.^27^ This parameter was decreased after both the first and the second test, demonstrating the effort performed after a submaximal exercise, accompanied by an increase in total (R5) and central (R20) respiratory resistance.

In this sense, this study on pulmonary function assessed by spirometry before and after the 6MWT showed that this test does not cause a decrease in lung volumes or capacities, which affirms its safe applicability in the sample; however, a significant decrease in the spirometric variable FEF_25-75%_ was seen. This variable represents the mean forced expiratory flow of a segment during forced vital capacity maneuver (FVC), which includes the airway flow of medium and small gauges and is considered a sensitive parameter to detect small airway obstruction.^28^ ^,^ ^29^ This response after the 6MWT may be analogous to bronchoconstrictive effect of small airways after exercise.^29^ Fonseca et al.^30^ discussed this effect in a study using spirometry after bronchoprovocation by exercise in children with ashtma of varying levels of severity. They found that this biomarker may be reduced in response to exercise, especially in children with mild asthma, and recommended its use, along with first-second forced expiratory volume (FEV_1_), as a criterion for bronchoconstricting response to exercise.^29^

This study also analyzed IOS response to 6MWT and identified a significant increase in R5 after the test (p=0.028). IOS has been used to analyze the effects of procedures and therapeutics. In this sense, Lee et al.^31^ evaluated the characteristics of airway obstruction in 47 asthmatic young patients (mean age 20.7 years) after a bronchoprovocation test with treadmill methacholine, and reported that R5 increased in the group showing hyperresponsive airways at 5 and 10 minutes after the end of the test. The authors concluded that IOS may be useful for objective assessments and to improve understanding of exercise-induced airway obstruction in young asthmatics. This finding places IOS as a useful tool for early diagnosis of asthma. In our research, increase in R5 after 6MWT can also be justified by the instability of small airways during physical effort, although it is a relatively fast stimulus and involves submaximal effort.

In addition to asthma, IOS has been considered relevant for cystic fibrosis. Vendrusculo et al.^32^ revealed a relationship between respiratory muscle strength and lung mechanics in this population, while Díez et al.^33^ found correlations between FEV_1_ and some oscillometric parameters (Z, R5, X5). Moreau et al.^34^ had already identified an inverse relationship between R5, Z and Fres values and spirometric parameters such as FEV_1_ in 15 children with cystic fibrosis aged 4 to 19 years.

Complementarily, IOS and spirometry have been recently used to assess lung function of 2,621 Swedish school-age children (149 preterm and 2,472 at term). The authors observed that at 8 years of age FEV_1_ was lower in preterm *versus* term females, but not in preterm males. For the preterm group, IOS showed increased resistance adjusted at 5 Hz for males compared to the same term group.^35^

Studies using these both instruments and relating them to exercise testings, likewise the present research, as well as evaluating healthy populations are scarce. IOS has been used as a tool to identify and monitor respiratory system changes due to dysfunctions such as asthma and cystic fibrosis, which makes it difficult to compare and establish normality parameters. In this context, studies on the understanding and use of respiratory resistance measurements arre still infrequent and require clarification.

Regarding these findings, the proportion of overweight and obese children was checked and, after detailed statistical analysis, no difference was found between spirometric and oscillometric values between participants. Therefore, we chose not to create groupings associated with body mass. As for ethnicity, all participnts were classified as White. The literature lacks studies that correlate ethnic differences with performance in the 6MWT, even although pulmonary function is known to be influenced by this characteristic.

To ensure internal and research validity, a strict caution in the control of health history and prematurity was taken in sample selection, in addition to the verification of absence of pulmonary diseases and spirometric values according to normality, so that participants of both groups would not interfere with these factors. Also, possible bias regarding the repercussion of forced expiratory maneuvers was avoided, as inclusion criterias was limited to children who did not exceed 12 maneuvers. Although the sample was small, it reached the previous sample calculation and, with the possible biases under control, it seems to have been representative of healthy schoolchildren. The selection of participants and inclusion criteria, such as maximum number of forced expiratory maneuvers, made it possible to control the external validity of the study, as well as the absence of a relationship between 6MWT and students being considered eutrophic or not.

The importance of this study is the possibility of clarifying the relationship between IOS and other evaluation instruments, especially those already established in the scientific community, such as spirometry and the 6MWT. Multicentric studies with separate individuals grouped by gender, weight and age, height and ethnicity, are necessary to achieve accuracy of results found by the present study. Thus, understanding physiological and respiratory responses to physical effort should be guided by studies with larger samples.

The present study examined respiratory mechanics and lung function in children and adolescents submitted to the 6MWT and identified an increase in airway resistance (total and central), as well as a significant decrease in FEF_25-75%_ after the test. These findings point to more attention and caution when performing submaximal tests in children presenting with predisposition to airway abnormalities, especially when undergoing exercise testings, as performed with children with asthma and cystic fibrosis.
